# Loneliness among Rural and Underserved Older Adults During the COVID-19 Pandemic

**DOI:** 10.1093/geroni/igaa057.3480

**Published:** 2020-12-16

**Authors:** Lindsay Wilkinson, Julie Masters, Christopher Kelly, Miechelle McKelvey, Ladan Ghazi Saidi, Toni Hill

**Affiliations:** 1 University of Nebraska Omaha, Omaha, Nebraska, United States; 2 University of Nebraska Omaha, Lincoln, Nebraska, United States; 3 University of Nebraska Kearney, Kearney, Nebraska, United States; 4 University of Nebraska - Kearney, Kearney, Nebraska, United States

## Abstract

During the COVID-19 pandemic, older adults are among the most vulnerable populations to the medical complications of COVID-19; however, they are also deeply affected by the unintended consequences of social distancing and sheltering in place. Social distancing effectively mitigates the spread of COVID-19, but this practice can also lead to social isolation and loneliness. Drawing on a sample of adults age 60 or older receiving Meals on Wheels/Grab and Go Meals in the state of Nebraska, this study investigates loneliness among rural and underserved older adults during the COVID-19 pandemic. Surveys were distributed to 3725 meal recipients across Nebraska’s eight Area Agencies on Aging in July 2020 (response rate = 50%), and a stratified random subsample was selected for preliminary analysis (N = 240). Logistic regression models were used to estimate the effects of COVID-19 and its associated safety precautions on loneliness. The findings reveal that 1 in 10 older adults have not left their home in over a month, and 38 percent feel lonelier due to the impact of COVID-19. Older adults who engaged in more community activities before the pandemic, reported leaving their home less, and experienced a longer absence of social interaction since the pandemic all had significantly increased odds of feeling lonelier in the COVID-19 era. Longer duration of sheltering in place was marginally associated with increased loneliness. The findings from this study show the consequences of social distancing on rural and underserved older adults, which calls for coordinated intervention.

